# Crystallographic, vibrational modes and optical properties data of α-DIPAB crystal

**DOI:** 10.1016/j.dib.2017.11.074

**Published:** 2017-11-26

**Authors:** Ahmad Alsaad, Chris M. Marin, Nabil Alaqtash, Hsien-Wen Chao, Tsun-Hsu Chang, Chin Li Cheung, A. Ahmad, I.A. Qattan, Renat F. Sabirianov

**Affiliations:** a Department of Physical Sciences, Jordan University of Science and Technology, P.O. Box 3030, Irbid 22110, Jordan; b Department of Chemistry, University of Nebraska-Lincoln, Lincoln, NE 68588, United States; c Department of Physics, The Hashemite University, P.O. Box 330127, Zarqa 13133, Jordan; d Department of Physics, National Tsing Hua University, Hsinchu 300, Taiwan; e Department of Physics, University of Nebraska at Omaha, Omaha, NE 68182, United States; f Department of Physics, Khalifa University of Science and Technology, P.O. Box 127788, Abu Dhabi, United Arab Emirates

**Keywords:** Powder X-ray diffraction, Density functional theory, Van der Waals interactions, Vibrational modes, Bromine deficient samples, GGA approximation, HSE06 hybrid functionals

## Abstract

The Crystallographic data of the α-DIPAB sample was measured using powder X-ray diffraction (PXRD). The crystal structure was also optimized using density functional based method. The calculations were performed both including van der Waals (vdW) interactions and excluding them to quantify the effects of vdW interaction on the crystal formation. The vibrational modes of DIPAB crystal corresponding to the Bromine deficient samples are calculated and presented. These show the origin of drastic change in dielectric response in Br deficient samples as compared to the ideal stoichiometric DIPAB crystal (Alsaad et al. 2018) [4]. Optical properties of an idealα-DIPAB were calculated using GGA and HSE06 hybrid functional methods implemented in VASP package. We mainly calculated the real and imaginary parts of the frequency-dependent linear dielectric function, as well as the related quantities such as the absorption, reflectivity, energy-loss function, and refractive index of α-DIPAB in the energy window of (0–12) eV.

**Specifications Table**

**Table:** 

Subject area	Physics, Chemistry
More specific subject area	Molecular Ferroelectric Crystals
Type of data	Table, (x-ray diffraction), graph, figure
How data was acquired	The crystallographic data of the α-DIPAB sample was measured using powder X-ray diffraction (PXRD).The crystal structure was also optimized using density functional based method. The calculations were performed both including van der Waals (vdW) interactions and excluding them to quantify the effects of vdW interaction on the crystal formation. The vibrational modes of DIPAB crystal corresponding to the Bromine deficient samples are calculated and presented using density functional based theory. Optical properties of an ideal α-DIPAB were calculated using GGA and HSE06 hybrid functional methods implemented in VASP package.
Data format	Raw data is analyzed
Experimental factors	Theα-phase diisopropylammonium bromide (α-DIPAB) was synthesized by the direct reaction of diisopropylamine (99.5% DIPA, Acros Organics) with hydrobromic acid (47–49% HBr, Fisher). Both reagents were used without further purification. Briefly, in a typical reaction, 4 mL of HBr was rapidly added to 5 mL of DIPA in an open beaker chilled in an ice bath. Upon addition, white precipitate immediately formed. Once the mixture cooled down, 70 mL of dry methanol (99.9%, Fluka) was used to re-dissolve the white powder with vigorous stirring and heating to 30 °C. The methanol solution was placed in a freezer to recrystallize the product overnight. The obtained long, needle-like crystals were then separated from the solution and dried in a convection oven at 50 °C for 1.5 h. Finally, to produce pure α-DIPAB products, 0.5 g of the white crystals were annealed in a commercial microwave oven operated at 700 W for 15 s, resulting in a fine white powder.
Experimental features	The crystal structure of theα-DIPAB sample was assessed using powder X-ray diffraction (PXRD). The Cu K*α* source of the diffractometer (PANalytical Empyrean) has an average wavelength of 1.544 Å. The FT-IR spectrum of the sample was obtained using a Thermo Nicolet Avatar 380 FT-IR with a Smart Performer ATR accessory and a zinc selenide crystal. The Raman spectroscopy was performed using a DXR Raman microscope (Thermo Scientific) with a 532 nm laser. The dielectric property and loss tangent measurements of pelleted α-DIPAB samples were performed using a home-made resonant cavity at 2.45 GHz.
Data source location	Omaha, Lincoln, Nebraska, USALongitude of Omaha (NE): −95.9979883, Latitude of Omaha (NE): 41.2523634, Latitude of Linclon (NE): 40.806862 DMS Lat: 40° 48′ 24.7032″ N, Longitude of Linclon (NE): −96.681679 DMS Long: 96° 40′ 54.0444″ W
Data accessibility	Data available in this article

**Value of the data**

• The data represents experimental preparation and characterization of α-DIPAB samples. We present the details of chemical preparation of the samples, crystallographic data and vibrational modes of an idealα-DIPAB polar crystal, as well as, those of Br-deficient DIPAB.
• Optical properties data of an ideal α-DIPAB is reported using DFT-based calculations. Specifically, GGA and the hybrid HSE06 functionals were used in obtaining the real and imaginary parts of the frequency-dependent linear dielectric function, as well as therelated quantities such as the absorption, reflectivity, energy-loss function, and refractive index of α-DIPAB in the energy window of (0–12) eV.
• The PXRD spectra suggest overall excellent crystallinity. However, the vibrational spectra, investigated by FT-IR and Raman spectroscopy, indicate the presence of Br disorder. Our DFT-based calculations show that Br vacancies drastically alter the strength and nature of crystal bonding, as well as vibrational spectra and dielectric constant of the material.
• Our data offers an open invitation for other researchers from chemical and physical communities who are interested in molecular ferroelectric crystals, in general, and DIPAB crystal, in particular, to collaborate to dig deeper into structural, chemical and physical properties of this novel polar molecular crystal.
• As a result, we believe that our data could be potentially used for designing future experiments and that our theoretical model could serve as a benchmark for future computational models that could be used to initiate new experiments on α-DIPAB crystal.
• We found an excellent agreement between experimental data and computational data on structural, dielectric and lattice dynamical properties of α-DIPAB.

## 1 Data

**Fig. 1:**
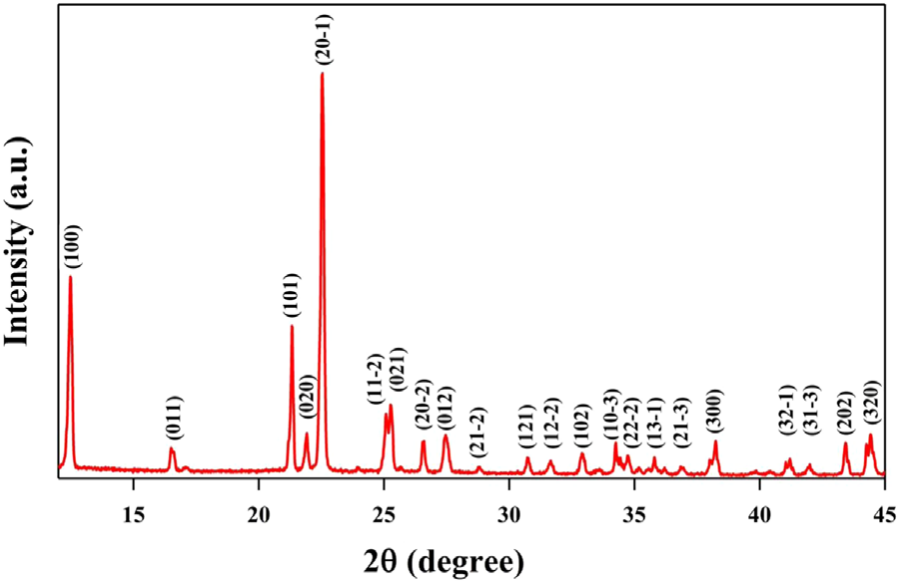
PXRD pattern of our synthesized DIPAB polycrystalline sample. Peaks correspond to α-phase of DIPAB.

**Fig. 2:**
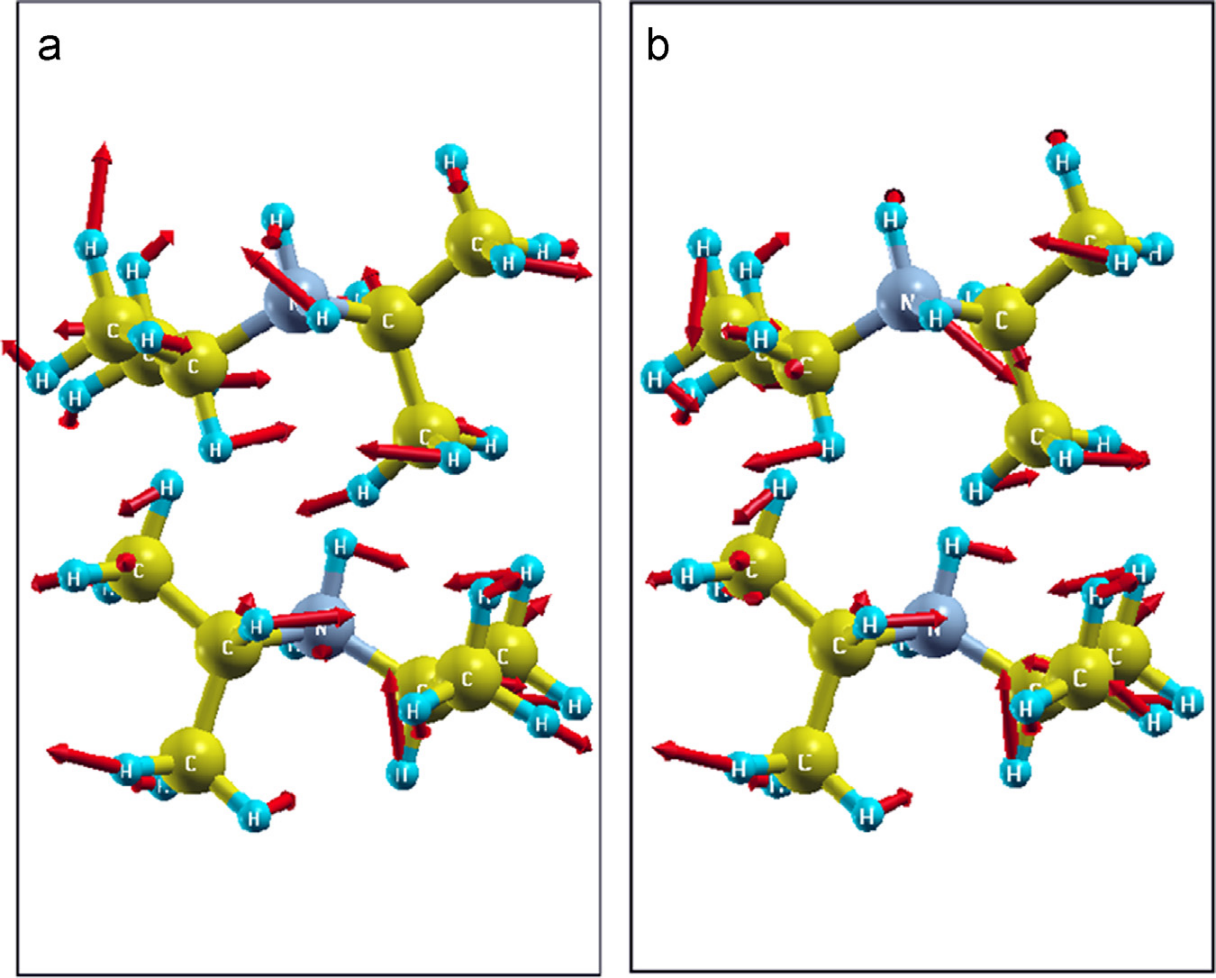
Eigenvectors of the vibrational modes at (a) 182.95 cm^−1^ and (b) 181.41 cm^−1^, respectively. Red arrows indicate the vibrational modes for different atoms.

The data set used in this Data Brief article involves the crystallographic data of α-DIPAB molecular ferroelectric crystal obtained using XRD-derived structural parameters as the initial structure in our *ab initio* calculations (see Table 1).The powder x-ray diffraction (PXRD pattern indicates that the α-DIPAB sample has an overall excellent crystallinity (see Fig. 1)).The crystal structure of DIPAB obtained by PXRD agrees well with the previously reported data [1], [2], [3]. However, our analysis of FT-IR and Raman vibrational spectra of α-DIPAB suggests the presence of disorder in the synthesized crystals as indicated by the presence of broad features in the Raman spectrum (See Fig. 1(c) and (d) in Ref. [4]). We have used the first-principles based vdW+DF2 calculations to pinpoint the vibrational modes in the Raman spectra and examined the ones due to Br-disorder. Our computational data reveals the strong dependency of dielectric constant of α-DIPAB on the bromine (Br) deficiency. Furthermore,including the van der Waals forces in our DFT-based calculations have a slight effect on the structural parameters and causing a small shift in the vibrational frequencies. Our Raman spectra and DFT-based data indicate that vibrational modes of the DIPAB crystal in the Raman spectrum were driven by covalent bonding in the DIPA molecules and hydrogen bonds.The Br-mediated hydrogen bond is strong and stabilizes *α*-phase. Fig. 2 illustrates the eigenvectors of the vibrational modes of the Br-deficient DIPAB at (a) 182.95 cm^−1^ and (b) 181.41 cm^−1^, respectively. The vibrational modes of different atoms are indicated by the red arrows the vibrational modes for different atoms.

**Table 1:** Structural properties and spontaneous polarization of the centrosymmetric monoclinic *P*2_1/m_ (*β*) and polar monoclinic *P*2_1_ (*α*) phases of DIPAB ferroelectric molecular crystal. Bracketed values correspond to values calculated using the vdW+DF2 method.

Space group	*P*2_1/m_ (*β*)	*P*2_1_ (*α*)
Empirical formula	C_6_ H_16_ Br N	C_6_ H_16_ Br N
Lattice parameter, *a* (Å)	7.946±0.09	7.799±0.1 (7.954)
Lattice parameter, *b* (Å)	8.1567±0.06	8.067±0.08 (8.227)
Lattice parameter, *c* (Å)	7.974±0.08	7.046±0.07 (7.188)
α (deg)	90	90
β (deg)	116.511±0.23	116.231±0.30
γ (deg)	90	90
Equilibrium volume, *V* (Å^3^)	463.05	443.30 (470.43)
Spontaneous polarization, *P*_*s*_ (μC/cm^2^)	0	22.64

Table 2 lists the vibrational frequencies that mainly appear in an ideal DIPAB and those appear after Br is removed. the vibration modes frequencies of an ideal DIPAB are determined primarily by the covalent bonding between atoms of DIPA molecules and hydrogen bonds of the molecules with Br. Modes specifically related to Br bonding with DIPA are at the wavenumbers of 213, 382, 515, 845 and 1615 cm^−1^, as indicated by the arrows in Fig. 1(c) of Ref. [1]. Table 3, Table 4, Table 5, Table 6, Table 7 lists the eigenvectors of the vibrational modes that appear in an ideal DIPAB. These vibrational frequencies disappear in calculated spectra of Br-deficient DIPAB (See Fig. 1(d) of Ref. [1]). The modification of the bonding in Br-deficient DIPAB is significantly exhibited by its considerable effect on the dielectric constant of DIPAB. The calculated static dielectric constant is only 2.3. Our measurements show a much larger value of the dielectric constant. The α-DIPAB sample has a measured dielectric constant of 17.4±0.25 at room temperature. Our DFT based calculations of static dielectric constant of the Br-deficient crystals yields a value of *ε*=18. This value is much closer to the experimentally measuredε. Obviously, This result is due to the appearance of a large number of low-energy excitation modes in the absence of strong Br-DIPA hydrogen bond that modifies the molecular dipole alignment in the crystal. Table 2 shows two of such modes. Mainly those appears at wavenumbers of 182.95 cm^−1^ and 181.41 cm^−1^. Table 8, Table 9, Table 10 list the eigenvectors of the vibrational modes that appear in Br-deficient DIPAB. Our DFT based data indicates that the eigenvectors of the vibrational modes at 182.95 cm^−1^ and 181.41 cm^−1^ correspond to twisting/rotation of the molecules as shown in Fig. 2. The changes in C-N-C angle and the corresponding shift of hydrogen atoms in this mode change the electric polarization of the molecules.

**Table 3:** Eigenvectors of the vibrational modes of an ideal DIPAB at 213 cm^−1^.

*X*	*Y*	*Z*	*dx*	*dy*	*dz*
−0.0772	6.96393	2.60214	0.00083	0.00472	−0.0023
4.47393	2.85027	4.58577	−0.0008	0.00472	0.00233
−0.3577	3.63793	3.4311	0.01758	−0.0664	0.00642
4.75446	7.75159	3.75681	−0.0176	−0.0664	−0.0064
0.61229	3.41086	4.62303	0.00862	−0.0248	0.02655
0.58545	1.93376	5.08133	0.03967	−0.0047	0.09331
0.19576	4.38814	5.75404	−0.0514	0.013	−0.0259
−0.16	2.81272	2.12702	0.00581	−0.0149	−0.0319
6.71175	3.27972	1.11979	−0.0074	0.06517	0.01459
1.27513	2.98791	1.57375	−0.0071	−0.0012	−0.0597
3.78449	7.52452	2.56488	−0.0086	−0.0248	−0.0266
3.81132	6.04742	2.10658	−0.0397	−0.0046	−0.0933
4.20101	0.27448	1.43387	0.05142	0.013	0.02594
4.55675	6.92638	5.0609	−0.0058	−0.0149	0.0319
−2.315	7.39338	6.06812	0.00744	0.06521	−0.0146
3.12164	7.10157	5.61416	0.00711	−0.0012	0.05971
−1.3409	3.45418	3.77369	0.01883	−0.0846	0.0025
−0.3099	4.66222	3.17472	−0.0055	−0.0632	0.0232
1.61909	3.67281	4.25278	0.00948	−0.017	0.03304
0.88237	1.23452	4.28036	0.03036	−0.0314	0.11153
−0.4264	1.65428	5.43492	0.05197	−0.0049	0.1272
1.2994	1.81691	5.91745	0.06107	0.04202	0.08121
0.19291	5.43422	5.39477	−0.0557	−0.0002	−0.0645
0.91631	4.30974	6.5873	−0.0837	0.05601	0.00597
−0.8148	4.13655	6.13394	−0.0617	0.00894	−0.0566
−0.3353	1.75739	2.4059	0.00203	−0.0265	−0.0745
5.6985	3.18373	1.55404	−0.0019	0.06413	0.02707
6.77518	2.66813	0.20297	−0.0367	0.1159	−0.0213
6.88107	4.33776	0.83491	0.01208	0.07763	0.07282
2.04424	2.59847	2.26477	0.00754	−0.0062	−0.0778
1.48512	4.05723	1.37189	−0.0158	0.00157	−0.0527
1.34872	2.43773	0.6168	−0.026	0.00958	−0.0674
5.73763	7.56784	3.41422	−0.0189	−0.0846	−0.0025
4.70664	0.54856	4.01319	0.00549	−0.0632	−0.0232
2.77769	7.78647	2.93514	−0.0095	−0.017	−0.0331
3.5144	5.34818	2.90755	−0.0304	−0.0314	−0.1116
4.82322	5.76794	1.75299	−0.052	−0.0049	−0.1272
3.09737	5.93057	1.27046	−0.0611	0.04203	−0.0812
4.20386	1.32056	1.79314	0.05574	−0.0002	0.06451
3.48046	0.19608	0.60061	0.08378	0.05603	−0.006
5.21161	0.02289	1.05398	0.06177	0.00894	0.05664
4.73208	5.87105	4.78202	−0.002	−0.0265	0.07455
−1.3017	7.29739	5.63387	0.00192	0.06417	−0.0271
−2.3784	6.78179	6.98494	0.03674	0.11595	0.02125
−2.4843	0.2241	6.353	−0.0121	0.07768	−0.0729
2.35253	6.71213	4.92314	−0.0075	−0.0063	0.07788
2.91165	8.17089	5.81602	0.01576	0.00154	0.05274
3.04805	6.55139	6.57111	0.02605	0.00955	0.06745

**Table 2:** The vibrational frequencies that appear in ideal DIPAB (with Br bonds) and vibrational frequencies that appear after Br is removed.

Vibrational frequencies with Br bonds (cm^−1^)	Intensity (a.u.)	Vibrational frequencies after Br is removed (cm^−1^)	Intensity (a.u.)
213	0.40	182.95	0.20
382	0.50	181.41	0.40
515	0.60		
845	1.10		
1615	1.20

**Table 4:** Eigenvectors of the vibrational modes of an ideal DIPAB at 382 cm^−1^.

*X*	*Y*	*Z*	*dx*	*dy*	*dz*
−0.0772	6.96393	2.60214	0.00166	0.00734	−0.0005
4.47393	2.85027	4.58577	−0.0016	0.00734	0.00051
−0.3577	3.63793	3.4311	−0.0612	−0.0914	0.27109
4.75446	7.75159	3.75681	0.06118	−0.0912	−0.2707
0.61229	3.41086	4.62303	0.25837	0.03895	−0.0111
0.58545	1.93376	5.08133	−0.0677	0.07289	−0.0352
0.19576	4.38814	5.75404	−0.0488	−0.0636	−0.0915
−0.16	2.81272	2.12702	0.02	0.26078	0.02036
6.71175	3.27972	1.11979	−0.0615	−0.0476	−0.0919
1.27513	2.98791	1.57375	0.06992	−0.0535	−0.0462
3.78449	7.52452	2.56488	−0.258	0.03889	0.01114
3.81132	6.04742	2.10658	0.06763	0.07278	0.03509
4.20101	0.27448	1.43387	0.04873	−0.0635	0.09134
4.55675	6.92638	5.0609	−0.02	0.2604	−0.0203
−2.315	7.39338	6.06812	0.06138	−0.0475	0.09176
3.12164	7.10157	5.61416	−0.0698	−0.0534	0.04612
−1.3409	3.45418	3.77369	0.02331	−0.1711	0.12091
−0.3099	4.66222	3.17472	−0.1608	−0.0054	0.13645
1.61909	3.67281	4.25278	0.0629	0.01855	−0.0267
0.88237	1.23452	4.28036	−0.0748	−0.0051	−0.0077
−0.4264	1.65428	5.43492	−0.0593	0.1212	−0.0407
1.2994	1.81691	5.91745	−0.0592	−0.0578	0.01173
0.19291	5.43422	5.39477	−0.0758	−0.0195	−0.0289
0.91631	4.30974	6.5873	−0.0681	0.03913	0.02481
−0.8148	4.13655	6.13394	−0.0214	−0.0947	−0.0947
−0.3353	1.75739	2.4059	0.01314	0.06679	−0.0189
5.6985	3.18373	1.55404	−0.0166	−0.0754	−0.0381
6.77518	2.66813	0.20297	0.04729	−0.067	0.0125
6.88107	4.33776	0.83491	−0.099	−0.018	−0.0881
2.04424	2.59847	2.26477	−0.0055	−0.0701	−0.0152
1.48512	4.05723	1.37189	0.12071	−0.0449	−0.0581
1.34872	2.43773	0.6168	−0.0514	−0.0632	0.00786
5.73763	7.56784	3.41422	−0.0233	−0.1708	−0.1207
4.70664	0.54856	4.01319	0.16057	−0.0054	−0.1362
2.77769	7.78647	2.93514	−0.0628	0.01853	0.02669
3.5144	5.34818	2.90755	0.07468	−0.0051	0.00766
4.82322	5.76794	1.75299	0.05922	0.12102	0.0406
3.09737	5.93057	1.27046	0.0591	−0.0577	−0.0117
4.20386	1.32056	1.79314	0.07565	−0.0194	0.02885
3.48046	0.19608	0.60061	0.06804	0.03907	−0.0248
5.21161	0.02289	1.05398	0.02138	−0.0946	0.09452
4.73208	5.87105	4.78202	−0.0131	0.06669	0.01885
−1.3017	7.29739	5.63387	0.01658	−0.0752	0.03806
−2.3784	6.78179	6.98494	−0.0472	−0.0669	−0.0125
−2.4843	0.2241	6.353	0.0988	−0.018	0.08791
2.35253	6.71213	4.92314	0.00548	−0.07	0.01519
2.91165	8.17089	5.81602	−0.1205	−0.0448	0.05802
3.04805	6.55139	6.57111	0.05129	−0.0631	−0.0079

**Table 5:** Eigenvectors of the vibrational modes of ideal DIPAB at 515 cm^−1^.

*X*	*Y*	*Z*	*dx*	*dy*	*dz*
−0.0772	6.96393	2.60214	0.00719	−0.001	−0.0045
4.47393	2.85027	4.58577	0.0072	0.00097	−0.0045
−0.3577	3.63793	3.4311	−0.0628	−0.0914	0.26862
4.75446	7.75159	3.75681	−0.0629	0.09149	0.26903
0.61229	3.41086	4.62303	0.25741	0.03837	−0.0166
0.58545	1.93376	5.08133	−0.067	0.07149	−0.0324
0.19576	4.38814	5.75404	−0.047	−0.0682	−0.0919
−0.16	2.81272	2.12702	0.02017	0.26041	0.02162
6.71175	3.27972	1.11979	−0.0569	−0.0457	−0.0889
1.27513	2.98791	1.57375	0.06583	−0.0524	−0.0451
3.78449	7.52452	2.56488	0.25779	−0.0384	−0.0166
3.81132	6.04742	2.10658	−0.0671	−0.0716	−0.0324
4.20101	0.27448	1.43387	−0.0471	0.06824	−0.092
4.55675	6.92638	5.0609	0.02019	−0.2608	0.02165
−2.315	7.39338	6.06812	−0.057	0.04578	−0.089
3.12164	7.10157	5.61416	0.06594	0.0525	−0.0452
−1.3409	3.45418	3.77369	0.02501	−0.1756	0.11959
−0.3099	4.66222	3.17472	−0.1653	−0.0035	0.13677
1.61909	3.67281	4.25278	0.06205	0.01923	−0.0289
0.88237	1.23452	4.28036	−0.0718	−0.0078	−0.0041
−0.4264	1.65428	5.43492	−0.0602	0.12187	−0.0422
1.2994	1.81691	5.91745	−0.0637	−0.0567	0.01683
0.19291	5.43422	5.39477	−0.071	−0.0189	−0.0246
0.91631	4.30974	6.5873	−0.0708	0.03109	0.02657
−0.8148	4.13655	6.13394	−0.0224	−0.0944	−0.0971
−0.3353	1.75739	2.4059	0.01328	0.0665	−0.0189
5.6985	3.18373	1.55404	−0.0149	−0.0726	−0.036
6.77518	2.66813	0.20297	0.04478	−0.0699	0.01557
6.88107	4.33776	0.83491	−0.0945	−0.0188	−0.0902
2.04424	2.59847	2.26477	−0.0081	−0.0754	−0.017
1.48512	4.05723	1.37189	0.12155	−0.0442	−0.0529
1.34872	2.43773	0.6168	−0.0533	−0.0595	0.00615
5.73763	7.56784	3.41422	0.02504	0.17589	0.11977
4.70664	0.54856	4.01319	−0.1656	0.00353	0.13698
2.77769	7.78647	2.93514	0.06214	−0.0193	−0.029
3.5144	5.34818	2.90755	−0.0719	0.00777	−0.0041
4.82322	5.76794	1.75299	−0.0603	−0.122	−0.0422
3.09737	5.93057	1.27046	−0.0638	0.05683	0.01684
4.20386	1.32056	1.79314	−0.0711	0.01893	−0.0247
3.48046	0.19608	0.60061	−0.0709	−0.0312	0.02661
5.21161	0.02289	1.05398	−0.0224	0.09454	−0.0972
4.73208	5.87105	4.78202	0.0133	−0.0666	−0.0189
−1.3017	7.29739	5.63387	−0.0149	0.07268	−0.036
−2.3784	6.78179	6.98494	0.04485	0.06998	0.01559
−2.4843	0.2241	6.353	−0.0947	0.01878	−0.0903
2.35253	6.71213	4.92314	−0.0081	0.07546	−0.017
2.91165	8.17089	5.81602	0.12173	0.04432	−0.053
3.04805	6.55139	6.57111	−0.0534	0.05958	0.00616

**Table 6:** Eigenvectors of the vibrational modes of ideal DIPAB at 845 cm^−1^.

*X*	*Y*	*Z*	*dx*	*dy*	*dz*
−0.0772	6.96393	2.60214	0.00024	−0.0004	−8E-05
4.47393	2.85027	4.58577	0.00024	0.00045	−8E-05
−0.3577	3.63793	3.4311	−0.0023	0.04236	0.0457
4.75446	7.75159	3.75681	−0.0022	−0.0432	0.04678
0.61229	3.41086	4.62303	−0.0086	−0.0008	0.00763
0.58545	1.93376	5.08133	−0.0017	0.00606	0.00136
0.19576	4.38814	5.75404	0.00522	−0.0141	−0.0135
−0.16	2.81272	2.12702	0.01334	−0.0014	−0.1181
6.71175	3.27972	1.11979	0.06669	−0.0243	0.03759
1.27513	2.98791	1.57375	−0.0696	−0.0057	0.00328
3.78449	7.52452	2.56488	−0.0089	0.00069	0.00741
3.81132	6.04742	2.10658	−0.0017	−0.006	0.00138
4.20101	0.27448	1.43387	0.00523	0.01414	−0.0136
4.55675	6.92638	5.0609	0.01362	0.00148	−0.1205
−2.315	7.39338	6.06812	0.068	0.02477	0.03832
3.12164	7.10157	5.61416	−0.071	0.00585	0.00335
−1.3409	3.45418	3.77369	0.0083	−0.0254	0.03013
−0.3099	4.66222	3.17472	−0.0474	0.05027	0.07454
1.61909	3.67281	4.25278	0.00021	−0.0098	0.02346
0.88237	1.23452	4.28036	0.01202	0.01759	−0.004
−0.4264	1.65428	5.43492	0.00711	−0.0196	0.00463
1.2994	1.81691	5.91745	0.00764	0.01887	−0.0044
0.19291	5.43422	5.39477	0.00296	−0.0246	−0.0449
0.91631	4.30974	6.5873	−0.0056	0.01673	−0.0013
−0.8148	4.13655	6.13394	−0.0026	0.00053	−0.0233
−0.3353	1.75739	2.4059	0.0106	0.00533	−0.0923
5.6985	3.18373	1.55404	0.1189	−0.0648	0.14993
6.77518	2.66813	0.20297	−0.0316	−0.0078	0.01802
6.88107	4.33776	0.83491	0.00336	−0.0132	0.04326
2.04424	2.59847	2.26477	−0.1785	−0.0281	0.11067
1.48512	4.05723	1.37189	−0.0214	−0.0069	0.05022
1.34872	2.43773	0.6168	0.06501	−0.0074	0.01356
5.73763	7.56784	3.41422	0.00867	0.02605	0.03095
4.70664	0.54856	4.01319	−0.0484	−0.0513	0.07616
2.77769	7.78647	2.93514	0.00018	0.00991	0.02363
3.5144	5.34818	2.90755	0.01221	−0.0173	−0.0038
4.82322	5.76794	1.75299	0.00726	0.02003	0.00488
3.09737	5.93057	1.27046	0.00774	−0.0194	−0.0044
4.20386	1.32056	1.79314	0.0028	0.02469	−0.0453
3.48046	0.19608	0.60061	−0.0057	−0.0169	−0.0014
5.21161	0.02289	1.05398	−0.0026	−0.0004	−0.0236
4.73208	5.87105	4.78202	0.01083	−0.0054	−0.0942
−1.3017	7.29739	5.63387	0.12122	0.06603	0.15283
−2.3784	6.78179	6.98494	−0.0322	0.00804	0.01839
−2.4843	0.2241	6.353	0.00345	0.01342	0.04409
2.35253	6.71213	4.92314	−0.1821	0.02861	0.11292
2.91165	8.17089	5.81602	−0.0219	0.00705	0.05126
3.04805	6.55139	6.57111	0.06636	0.00756	0.01385

**Table 7:** Eigenvectors of the vibrational modes of ideal DIPAB at 1615 cm^−1^.

*X*	*Y*	*Z*	*dx*	*dy*	*dz*
−0.0772	6.96393	2.60214	−0.0031	−0.0015	0.00054
4.47393	2.85027	4.58577	−0.0031	0.00152	0.00054
−0.3577	3.63793	3.4311	−0.118	0.10818	0.01183
4.75446	7.75159	3.75681	−0.1179	−0.1082	0.01183
0.61229	3.41086	4.62303	−0.0151	0.01779	0.00089
0.58545	1.93376	5.08133	0.00055	−0.006	0.00769
0.19576	4.38814	5.75404	−0.003	−0.0003	−0.0031
−0.16	2.81272	2.12702	−0.0201	0.01337	0.00306
6.71175	3.27972	1.11979	−0.0001	0.00372	0.00361
1.27513	2.98791	1.57375	0.00677	0.0037	−0.005
3.78449	7.52452	2.56488	−0.0151	−0.0178	0.00087
3.81132	6.04742	2.10658	0.00055	0.00601	0.0077
4.20101	0.27448	1.43387	−0.003	0.00028	−0.0031
4.55675	6.92638	5.0609	−0.0201	−0.0134	0.00304
−2.315	7.39338	6.06812	−0.0001	−0.0037	0.00362
3.12164	7.10157	5.61416	0.00677	−0.0037	−0.005
−1.3409	3.45418	3.77369	0.09635	−0.4701	0.07545
−0.3099	4.66222	3.17472	0.46193	−0.0408	−0.1336
1.61909	3.67281	4.25278	−0.0026	−0.0036	0.0002
0.88237	1.23452	4.28036	−0.0066	−0.0048	0.00139
−0.4264	1.65428	5.43492	−0.0115	0.00295	−0.0225
1.2994	1.81691	5.91745	0.01499	0.01042	−0.0101
0.19291	5.43422	5.39477	0.01997	−0.0012	−0.0023
0.91631	4.30974	6.5873	−0.0045	0.00767	0.00464
−0.8148	4.13655	6.13394	0.00656	−0.0166	0.00559
−0.3353	1.75739	2.4059	−0.0016	0.00424	0.00237
5.6985	3.18373	1.55404	0.00323	−0.0254	0.00061
6.77518	2.66813	0.20297	−0.0108	0.00591	−0.0046
6.88107	4.33776	0.83491	0.02077	−0.0057	−0.0087
2.04424	2.59847	2.26477	0.00229	−0.0039	−0.0041
1.48512	4.05723	1.37189	−0.0084	0.0073	0.0136
1.34872	2.43773	0.6168	−0.0033	−0.0155	0.00828
5.73763	7.56784	3.41422	0.09632	0.47002	0.07543
4.70664	0.54856	4.01319	0.46182	0.04081	−0.1336
2.77769	7.78647	2.93514	−0.0026	0.00355	0.00021
3.5144	5.34818	2.90755	−0.0066	0.0048	0.00139
4.82322	5.76794	1.75299	−0.0115	−0.0029	−0.0226
3.09737	5.93057	1.27046	0.01501	−0.0104	−0.0101
4.20386	1.32056	1.79314	0.01995	0.00118	−0.0023
3.48046	0.19608	0.60061	−0.0045	−0.0077	0.00464
5.21161	0.02289	1.05398	0.00655	0.01661	0.00558
4.73208	5.87105	4.78202	−0.0016	−0.0043	0.00238
−1.3017	7.29739	5.63387	0.00324	0.02541	0.00061
−2.3784	6.78179	6.98494	−0.0108	−0.0059	−0.0046
−2.4843	0.2241	6.353	0.02078	0.00575	−0.0087
2.35253	6.71213	4.92314	0.00229	0.00388	−0.0041
2.91165	8.17089	5.81602	−0.0084	−0.0073	0.0136
3.04805	6.55139	6.57111	−0.0033	0.01549	0.00827

**Table 8:** Eigenvectors of the vibrational modes of ideal DIPAB at 182.95 cm^−1^.

*X*	*Y*	*Z*	*dx*	*dy*	*dz*
−0.3628	3.64199	3.43386	0.04116	−0.0398	−0.0011
4.75962	7.75565	3.75405	−0.041	−0.0397	0.00104
0.59804	3.40558	4.61605	0.012	−0.0182	0.02762
0.58428	1.94313	5.07252	0.02204	−0.0022	0.07713
0.19884	4.37336	5.74078	−0.0651	0.00976	−0.0212
−0.1517	2.83798	2.13711	0.01489	−0.006	−0.027
6.72813	3.28322	1.13375	−0.0115	0.06712	0.03037
1.26858	2.99581	1.58677	−0.0033	−0.0126	−0.0741
3.79873	7.51924	2.57187	−0.0119	−0.0182	−0.0275
3.81249	6.05679	2.11539	−0.0219	−0.0022	−0.0768
4.19793	0.2597	1.44713	0.06481	0.00971	0.0211
4.54844	6.95164	5.0508	−0.0148	−0.0059	0.02686
−2.3314	7.39688	6.05417	0.01145	0.06687	−0.0303
3.12819	7.10947	5.60114	0.00327	−0.0125	0.07379
−1.3481	3.43903	3.77366	0.03836	−0.0419	−0.0108
−0.3384	4.67325	3.19576	0.0388	−0.0377	0.01038
1.59649	3.66927	4.24733	0.02145	−0.0145	0.05337
0.88437	1.24395	4.28386	−0.0101	−0.0278	0.08679
−0.411	1.65387	5.42735	0.03552	0.01209	0.12508
1.29588	1.84017	5.90209	0.05517	0.02617	0.0525
0.20536	5.41149	5.38528	−0.0295	0.00252	−0.0432
0.91746	4.27911	6.56397	−0.1376	0.02121	0.04307
−0.8039	4.13916	6.11937	−0.0998	0.02474	−0.1028
−0.3313	1.78983	2.41259	0.00728	−0.0129	−0.0558
5.72328	3.17072	1.56385	−0.0006	0.04784	0.05187
6.80695	2.66259	0.23059	−0.0427	0.13328	−0.0173
6.87242	4.33056	0.84171	−0.0091	0.0876	0.10401
2.03382	2.60249	2.26655	0.01787	0.00566	−0.0865
1.49656	4.04457	1.37049	−0.0127	−0.0175	−0.1069
1.32947	2.43579	0.64261	−0.0335	−0.0356	−0.063
5.74491	7.55269	3.41426	−0.0382	−0.0418	0.01078
4.73519	0.55959	3.99216	−0.0386	−0.0376	−0.0104
2.80028	7.78293	2.94058	−0.0214	−0.0145	−0.0531
3.51241	5.35761	2.90405	0.01013	−0.0278	−0.0865
4.80778	5.76753	1.76056	−0.0354	0.01206	−0.1246
3.10089	5.95383	1.28582	−0.055	0.02604	−0.0523
4.19141	1.29783	1.80263	0.02934	0.00249	0.04309
3.47931	0.16545	0.62394	0.13699	0.02114	−0.0428
5.20071	0.0255	1.06854	0.09936	0.02464	0.10236
4.72803	5.90349	4.77532	−0.0073	−0.0128	0.05562
−1.3265	7.28438	5.62406	0.00055	0.04773	−0.0517
−2.4102	6.77625	6.95732	0.04257	0.13273	0.01722
−2.4756	0.2169	6.3462	0.00898	0.08725	−0.1036
2.36295	6.71615	4.92136	−0.0178	0.00559	0.08612
2.90021	8.15823	5.81742	0.01259	−0.0174	0.10627
3.0673	6.54945	6.54531	0.03331	−0.0353	0.0628

**Table 9:** Eigenvectors of the vibrational modes of DIPA at 182.95 cm^−1^.

*X*	*Y*	*Z*	*dx*	*dy*	*dz*
−0.3628	3.64199	3.43386	0.04116	−0.0398	−0.0011
4.75962	7.75565	3.75405	−0.041	−0.0397	0.00104
0.59804	3.40558	4.61605	0.012	−0.0182	0.02762
0.58428	1.94313	5.07252	0.02204	−0.0022	0.07713
0.19884	4.37336	5.74078	−0.0651	0.00976	−0.0212
−0.1517	2.83798	2.13711	0.01489	−0.006	−0.027
6.72813	3.28322	1.13375	−0.0115	0.06712	0.03037
1.26858	2.99581	1.58677	−0.0033	−0.0126	−0.0741
3.79873	7.51924	2.57187	−0.0119	−0.0182	−0.0275
3.81249	6.05679	2.11539	−0.0219	−0.0022	−0.0768
4.19793	0.2597	1.44713	0.06481	0.00971	0.0211
4.54844	6.95164	5.0508	−0.0148	−0.0059	0.02686
−2.3314	7.39688	6.05417	0.01145	0.06687	−0.0303
3.12819	7.10947	5.60114	0.00327	−0.0125	0.07379
−1.3481	3.43903	3.77366	0.03836	−0.0419	−0.0108
−0.3384	4.67325	3.19576	0.0388	−0.0377	0.01038
1.59649	3.66927	4.24733	0.02145	−0.0145	0.05337
0.88437	1.24395	4.28386	−0.0101	−0.0278	0.08679
−0.411	1.65387	5.42735	0.03552	0.01209	0.12508
1.29588	1.84017	5.90209	0.05517	0.02617	0.0525
0.20536	5.41149	5.38528	−0.0295	0.00252	−0.0432
0.91746	4.27911	6.56397	−0.1376	0.02121	0.04307
−0.8039	4.13916	6.11937	−0.0998	0.02474	−0.1028
−0.3313	1.78983	2.41259	0.00728	−0.0129	−0.0558
5.72328	3.17072	1.56385	−0.0006	0.04784	0.05187
6.80695	2.66259	0.23059	−0.0427	0.13328	−0.0173
6.87242	4.33056	0.84171	−0.0091	0.0876	0.10401
2.03382	2.60249	2.26655	0.01787	0.00566	−0.0865
1.49656	4.04457	1.37049	−0.0127	−0.0175	−0.1069
1.32947	2.43579	0.64261	−0.0335	−0.0356	−0.063
5.74491	7.55269	3.41426	−0.0382	−0.0418	0.01078
4.73519	0.55959	3.99216	−0.0386	−0.0376	−0.0104
2.80028	7.78293	2.94058	−0.0214	−0.0145	−0.0531
3.51241	5.35761	2.90405	0.01013	−0.0278	−0.0865
4.80778	5.76753	1.76056	−0.0354	0.01206	−0.1246
3.10089	5.95383	1.28582	−0.055	0.02604	−0.0523
4.19141	1.29783	1.80263	0.02934	0.00249	0.04309
3.47931	0.16545	0.62394	0.13699	0.02114	−0.0428
5.20071	0.0255	1.06854	0.09936	0.02464	0.10236
4.72803	5.90349	4.77532	−0.0073	−0.0128	0.05562
−1.3265	7.28438	5.62406	0.00055	0.04773	−0.0517
−2.4102	6.77625	6.95732	0.04257	0.13273	0.01722
−2.4756	0.2169	6.3462	0.00898	0.08725	−0.1036
2.36295	6.71615	4.92136	−0.0178	0.00559	0.08612
2.90021	8.15823	5.81742	0.01259	−0.0174	0.10627
3.0673	6.54945	6.54531	0.03331	−0.0353	0.0628

**Table 10:** Eigenvectors of the vibrational modes of DIPA at 181.41 cm^−1^.

*X*	*Y*	*Z*	*dx*	*dy*	*dz*
−0.3628	3.64199	3.43386	−0.0443	0.03372	0.0029
4.75962	7.75565	3.75405	−0.0445	−0.0339	0.0029
0.59804	3.40558	4.61605	−0.0136	0.01404	−0.0267
0.58428	1.94313	5.07252	−0.0238	0.00031	−0.0695
0.19884	4.37336	5.74078	0.0686	−0.01	0.02021
−0.1517	2.83798	2.13711	−0.0132	0.00342	0.02701
6.72813	3.28322	1.13375	0.01479	−0.0676	−0.0313
1.26858	2.99581	1.58677	0.00492	0.01998	0.07554
3.79873	7.51924	2.57187	−0.0136	−0.0141	−0.0268
3.81249	6.05679	2.11539	−0.0239	−0.0003	−0.0698
4.19793	0.2597	1.44713	0.06885	0.00999	0.02032
4.54844	6.95164	5.0508	−0.0132	−0.0034	0.02713
−2.3314	7.39688	6.05417	0.01484	0.06787	−0.0314
3.12819	7.10947	5.60114	0.00493	−0.02	0.07583
−1.3481	3.43903	3.77366	−0.0412	0.03615	0.01102
−0.3384	4.67325	3.19576	−0.0465	0.0329	−0.0067
1.59649	3.66927	4.24733	−0.0235	0.00716	−0.056
0.88437	1.24395	4.28386	−0.0079	0.01985	−0.08
−0.411	1.65387	5.42735	−0.0324	−0.0058	−0.0981
1.29588	1.84017	5.90209	−0.0424	−0.0275	−0.0573
0.20536	5.41149	5.38528	0.02657	−0.0039	0.03865
0.91746	4.27911	6.56397	0.14735	−0.017	−0.0492
−0.8039	4.13916	6.11937	0.10658	−0.0258	0.11014
−0.3313	1.78983	2.41259	−0.001	0.00888	0.05214
5.72328	3.17072	1.56385	0.00284	−0.0436	−0.0547
6.80695	2.66259	0.23059	0.04568	−0.1325	0.01555
6.87242	4.33056	0.84171	0.01678	−0.0884	−0.1035
2.03382	2.60249	2.26655	−0.0138	−0.0003	0.08422
1.49656	4.04457	1.37049	0.00815	0.02766	0.11646
1.32947	2.43579	0.64261	0.03757	0.05074	0.06002
5.74491	7.55269	3.41426	−0.0414	−0.0364	0.01106
4.73519	0.55959	3.99216	−0.0466	−0.0331	−0.0067
2.80028	7.78293	2.94058	−0.0236	−0.0073	−0.0562
3.51241	5.35761	2.90405	−0.0079	−0.02	−0.0804
4.80778	5.76753	1.76056	−0.0325	0.00582	−0.0986
3.10089	5.95383	1.28582	−0.0427	0.02755	−0.0576
4.19141	1.29783	1.80263	0.02668	0.0039	0.03887
3.47931	0.16545	0.62394	0.14789	0.01707	−0.0493
5.20071	0.0255	1.06854	0.10698	0.02593	0.11058
4.72803	5.90349	4.77532	−0.001	−0.0089	0.0524
−1.3265	7.28438	5.62406	0.00284	0.04391	−0.0549
−2.4102	6.77625	6.95732	0.04591	0.13309	0.01561
−2.4756	0.2169	6.3462	0.01678	0.08874	−0.1039
2.36295	6.71615	4.92136	−0.0139	0.0003	0.08457
2.90021	8.15823	5.81742	0.00817	−0.0277	0.11681
3.0673	6.54945	6.54531	0.03769	−0.0508	0.0603

DFT calculation show very good agreement with experimental structure both when vdW interactions were included and omitted. Taking vdW interaction into account increases the calculated lattice parameters up to 2%, as well as results in shift of the vibrational modes to larger wave number (See Figs (c-f) of Ref. [1]). The relatively modest effect of vdW interactions is due to the strong hydrogen bonds between Br and H atoms of DIPA molecules. These hydrogen bonds manifest themselves in the narrow peaks in the vibrational spectra with relatively high intensity. The removal of Br from the crystal results in disappearance of the above modes, while the lower wave number modes appear showing significantly higher rotational mobility of DIPA molecules in the crystal.

### 1.1 Optical properties of α-DIPAB

**Fig. 3:**
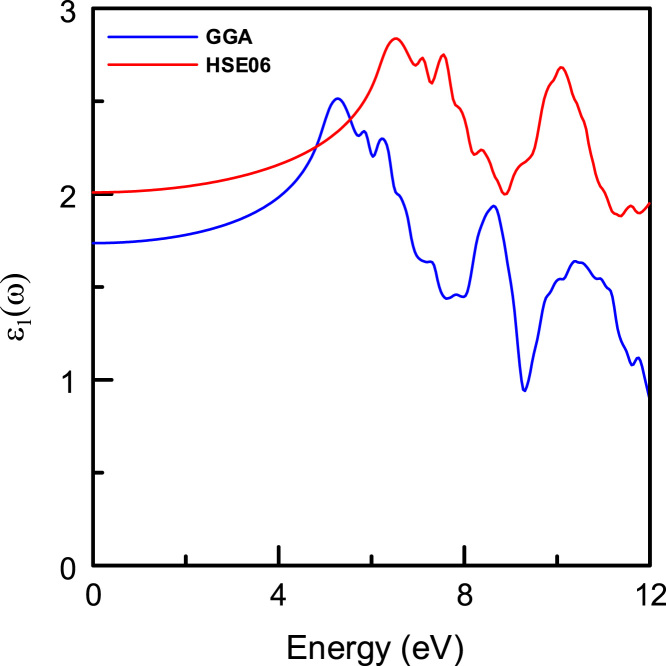
The real part $\varepsilon_{1}\left(\omega \right)$ of the complex dielectric function of α-DIPAB calculated using the GGA (Blue) and HSE06 Hybrid functional (Red) methods.

**Fig. 4:**
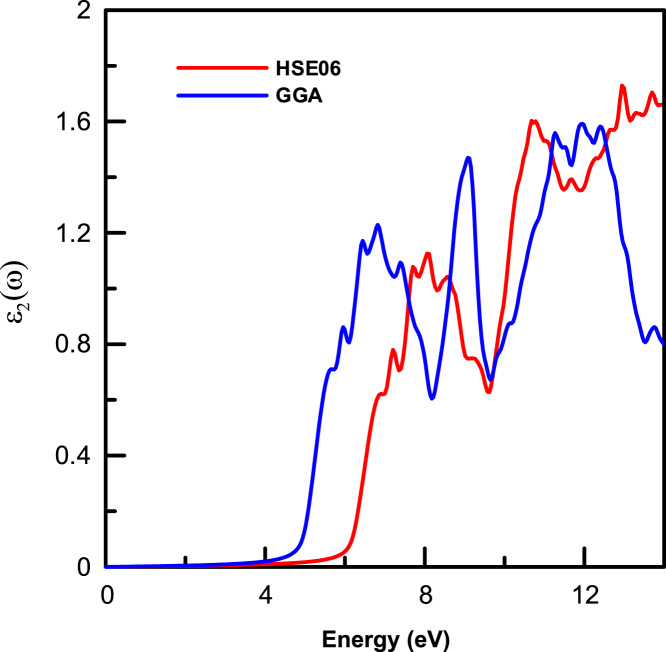
The imaginary part $\varepsilon_{2}\left(\omega \right)$ of the complex dielectric function of α-DIPAB calculated using the GGA (Blue) and HSE06 Hybrid functional (Red) methods.

**Fig. 5:**
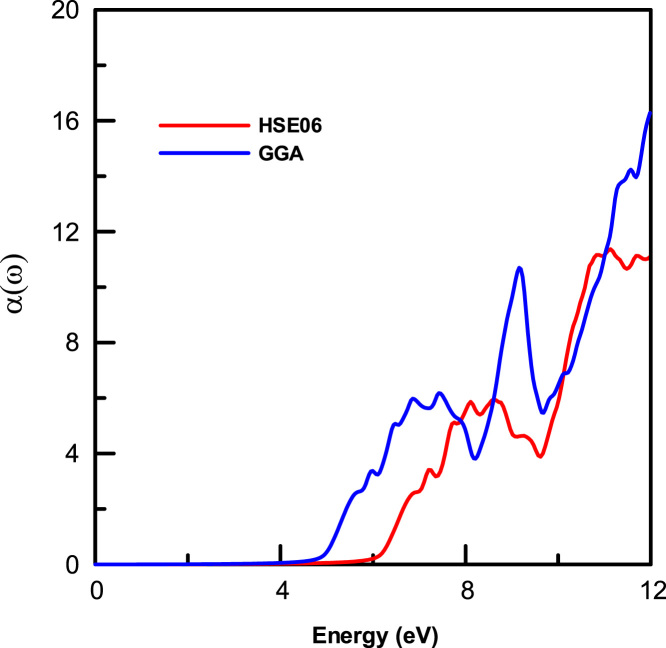
The absorption spectrum α(ω) of α-DIPAB calculated using the GGA (Blue) and HSE06 Hybrid functional (Red) methods.

**Fig. 6:**
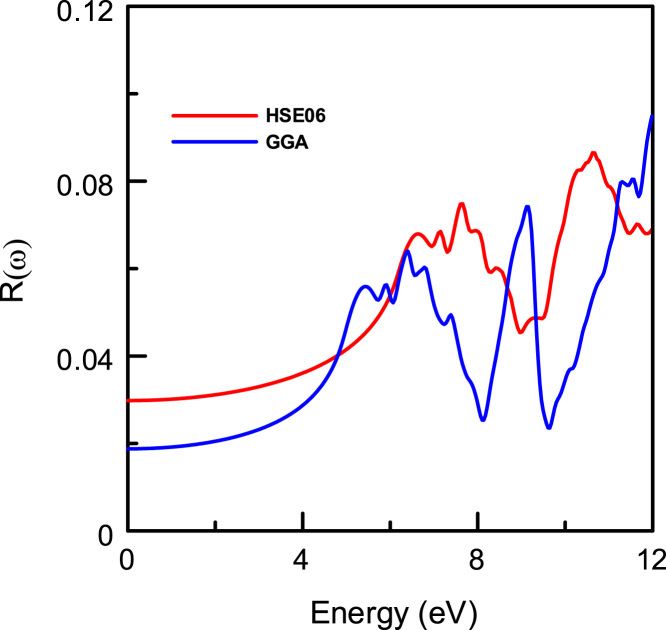
The reflectivity R(ω) of the dielectric function ofα-DIPAB molecular ferroelectric crystal using the GGA (Blue) and HSE06 Hybrid functional (Red) methods.

**Fig. 7:**
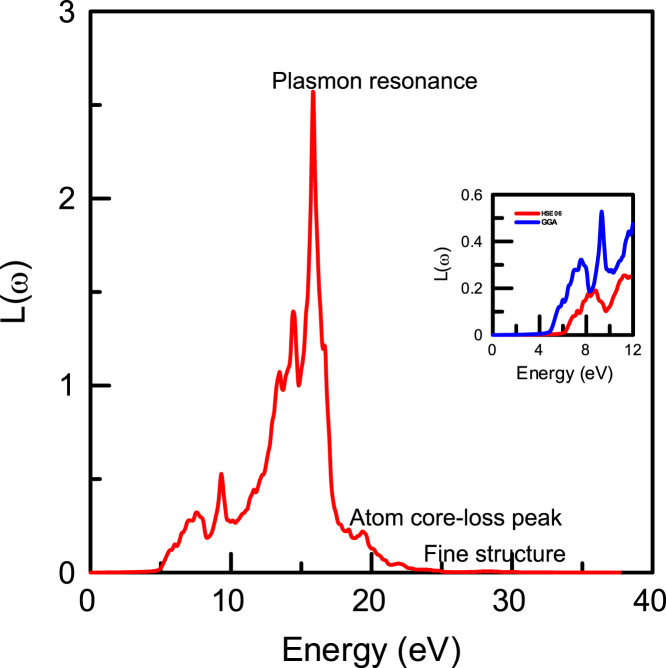
The energy-loss spectrum L(ω) of the dielectric function of α-DIPAB molecular ferroelectric crystal using the HSE06 Hybrid Functional method. The inset figure shows the energy-loss spectrum calculated using the GGA (Blue) and HSE06 Hybrid Functional (Red) methods in the range (0–12) eV.

**Fig. 8:**
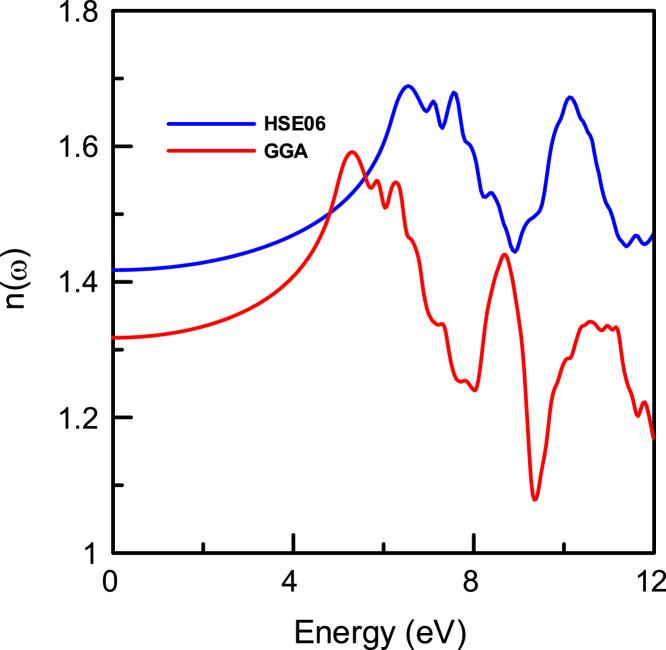
The real part of the refractive index n(ω) ofα-DIPAB using the GGA (Red) and HSE06 (Blue) methods.

**Fig. 9:**
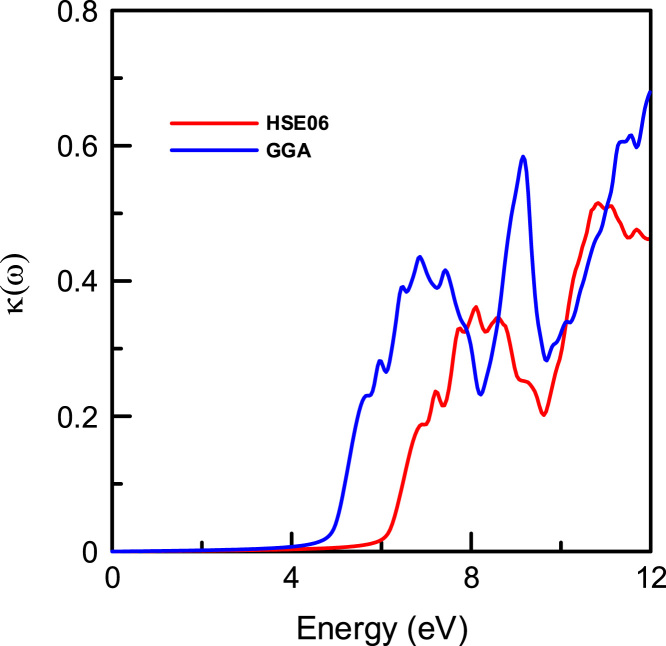
The imaginary part of the refractive index (extinction coefficient κ(ω)) ofα-DIPAB using the GGA (Blue) and HSE06 Hybrid functional (Red) methods.

Fig. 3, Fig. 4, Fig. 5, Fig. 6, Fig. 7, Fig. 8, Fig. 9 display optical properties of the α-DIPAB crystal calculated using the GGA and HSE06 hybrid functional methods. We have investigated the real and imaginary parts of the frequency-dependent linear dielectric function, as well as therelated quantities such as the absorption, reflectivity, energy-loss function, and refractive index of α-DIPAB in the energy window of (0–12) eV. Because the (0–12) eV photon energy range is sufficient to enrich our understanding of the spectral behavior of the optical functions, we have limited our investigation of the optical properties in this range. Fig. 3, Fig. 4 show the real and imaginary parts of the dielectric function as a function of the photon energy. Since the optical spectra have been analyzed for a wide photon energy range, the spectra contain two major peaks that occur at ~5 eV and ~8 eV which correspond to electronic transitions from the valence band of N and C atoms to the conduction bands of Br atoms. By analyzing Fig. 3, Fig. 4, Fig. 5, Fig. 6, Fig. 7, Fig. 8, Fig. 9, we found that the (0–4) eV photon energy range (using GGA method) and the (0–6) eV (using HSE06 hybrid functional method) are characterized by high transparency, no absorption (see Fig. 5), and small reflectivity (see Fig. 6). The calculated static dielectric constant $\varepsilon_{1}$(ω=0) of α-DIPABis found to be1.75 (2.00) using the GGA (HSE06) method.

As can be seen from Fig. 5, the absorption band of α-DIPAB starts at photon energy of 5 eV as predicted using GGA method and at 6 eV as calculated by HSE06 hybrid functionals. Above this absorption edge, the absorption peaks occur at 7 eV (9 eV) and 10 eV (11.5 eV) using the GGA (HSE06) method. Furthermore, α(ω) is shifted up to higher energy when calculated using the HSE06 hybrid functional. The ultraviolet-shift of the optical absorption peaks obtained by using the HSE06 hybrid functional method compared to those obtained using the GGA method indicates that the α-DIPABcould be used as a potential candidate in optoelectronic devices operating in the high energy range. Interestingly, α-DIPAB exhibits absorption coefficients that are of the same order of magnitude of TaO1_−x_N_1 +x_-based alloys [5].

Fig. 5 also indicates that incident light with energy less than 6 eV is not absorbed. On the other hand, incident light in energy range 6≤E≤12 eV is being absorbed. The steepest rise in α(ω) corresponds to the optical band gap occurs at ~ 5 eV (using GGA method) and at ~6 eV (using HSE06 hybrid functional method). The absorption coefficient, ***α***(ω), is related to the extinction coefficient κ by the following formula: α=4πκ/λ, where *λ* is the wavelength. Therefore, the extinction spectra ofα-DIPAB follow a similar spectral behavior to those of the absorption spectra as could be seen by comparing Figs. 5 and 9.

Fig. 6 shows that α-DIPABexhibits a reflectivity that is about an order of magnitude less than the reflectivity of TaO_1−x_N_1+x_-based alloys [5]. As can be seen from Fig. 6, the reflectivity R(ω) spectra exhibit two obvious reflection peaks located at 6 eV (8 eV) and 9 eV (11 eV) as obtained using the GGA (HSE06) method. The interband transition occurred mainly in the energy range of 8 eV ~9 eV (10 eV ~11.5 eV) as predicted by GGA and (HSE06) methods, respectively.

The sharp maxima in the energy-loss function L(ω) shown in Fig. 7 are associated with the existence of plasma oscillations ${\hbar \omega }_{p}$. Obviously, the calculated energy-loss function ofα-DIPAB indicates mainly sharp maxima peak at 15.82 eV. The peak value of volume loss, 15.82 eV, coincides with the zero value of the real part of the dielectric function. The inset in Fig. 7 shows the energy-loss spectrum calculated using the GGA and HSE06 methods in the energy range of (0–12) eV. Clearly, α-DIPABexhibits zero energy loss in the range (0–6) eV and negligible energy-loss in the energy window (6–12) eV. This result suggests that α-DIPABcould be used in devices that require minimal energy-loss. Optical transmission measurements can reveal information about the electronic band structure, fundamental optical band gap, and absorption from ionic lattice and molecules [6], [7], [8], [9], [10]. To obtain high transparency over an extended wavelength range, materials with low lattice resonance and high energy band gaps are required. Dielectric materials usually have large band-gap energy and only high energy light can excite the electronic structure. Because, the band gap of α-DIPABis ~(5–6) eV, this dielectric material tends to be transparent throughout the visible range. However, in the infrared range, the electromagnetic wave energy can excite the molecular vibrations and there can be an intrinsic absorption.

Fig. 8 shows the real part of the refractive index n(ω) as a function of the photon energy. The maximum value obtained at the edge of the optical band gap is 1.59 (1.69) using the GGA and (HSE06) method. The static refractive index n(ω=0) of ${P2}_{1}$ DIPAB is found to be 1.32 (GGA) and 1.42 (HSE06). Therefore, α-DIPAB has the potential to be used as a covering layer for several applications.

The extinction coefficient κ(ω) refers to several different measures of the absorption of light in a medium. It is closely related to the absorption function. From Fig. 9, one can see that the maximum value of κ(ω) of α-DIPABis 0.6 using GGA and 0.5 (using HSE06). This indicates an increase of the absorption of light for a given energy in polar phase of DIPAB. The maximum absorption occurs when a photon carries energy of about 9 eV is incident. This result paves the way for designing optoelectronic devices that work effectively at this value of energy.The main source of disagreement between optical results obtained using GGA and HSE06 hybrid functional is that the hybrid functional is based on screened coulomb potential and converges more rapidly with respect to the number of k-points as compared with GGA method. We recommend using HSE06 hybrid functional in calculating the optical properties of α-DIPAB.

## 2 Experimental design, materials and methods

The crystal structure of the α-DIPAB sample was assessed using powder X-ray diffraction (PXRD). The CuK*α* source of the diffractometer (PANalytical Empyrean) has an average wavelength of 1.544 Å. The FT-IR spectrum of the sample was obtained using a Thermo Nicolet Avatar 380 FT-IR with a Smart Performer ATR accessory and a zinc selenide crystal. The Raman spectroscopy was performed using a DXR Raman microscope (Thermo Scientific) with a 532 nm laser. The dielectric property and loss tangent measurements of pelleted α-DIPAB samples were performed using a home-made resonant cavity at 2.45 GHz.

## 3 Theoretical modeling of α-DIPAB molecular crystals

Density functional theory [11], [12] calculation of ideal and Br-deficient DIPAB crystal structures were used to calculate vibrational modes of the crystal. The projector augmented wave (PAW) method implemented in the VASP [13] with the Perdew-Burke-Ernzerhof (PBE) functional [14] for the exchange-correlation functional the cut-off energy is 400 eV and a 4×4×4 Monkhorst- pack grid. The atomic positions were relaxed with the Hellmann-Feynman forces <0.003 eV/Å. We applied a version of the van der Waals density functional (vdW-DF2) by Dion et al. [15] which employed a more accurate semi-local exchange functional and the use of a large-N asymptote gradient correction in determining the vdW kernel [16], [17]. We used the GGA [18] and hybrid functional HSE06 [19] methods to calculate the optical properties of α-DIPAB.
