# Supplementary material for: Crystallographic, vibrational modes and optical properties data of α-DIPAB crystal
Source: Data Brief. 2017 Nov 26;16:667–84. doi: 10.1016/j.dib.2017.11.074 (PMC5847493; doi:10.1016/j.dib.2017.11.074)
Supplement: Supplementary file 1 — Supplementary material [file mmc1.docx]

Conflicts of interests

'Conflicts of interest: none'
